# Dealing with difficult clients *via* personalized chaperone inhibitors

**DOI:** 10.1016/j.jbc.2020.100211

**Published:** 2021-01-28

**Authors:** Andrew W. Truman

**Affiliations:** Department of Biological Sciences, The University of North Carolina at Charlotte, Charlotte, North Carolina, USA

**Keywords:** CHIP, carboxy-terminus of Hsp70 interacting protein, CRPC, castration-resistant prostate cancer cells, RNR, ribonucleotide reductase

## Abstract

The importance of molecular chaperones in cancer is well established, yet several chaperone inhibitors have failed in clinical trials due to toxicity. Recent efforts have focused on targeting chaperone function in cancer by either manipulating the “chaperone code” or inhibiting helper cochaperones, such as DNAJA1. Tong *et al*. identify a novel inhibitor that specifically disrupts DNAJA1's interaction with p53, promoting p53 degradation. This finding highlights specific DNAJA1 interactions with the potential for less toxicity compared to traditional chaperone inhibitors.

Molecular chaperones are highly expressed proteins that act as custodians of the cell, helping to fold a large fraction of the proteome. The most well studied of these are Hsp70 and Hsp90, which are critical for cell viability (1). Hsp70 works at the initial stages of folding, binding newly synthesized and denatured proteins. In contrast, Hsp90 binds clients after they have been processed by Hsp70, promoting protein maturation and activation. Due to their ability to stabilize selected oncoproteins, significant research into development of chaperone-targeting anticancer therapeutics has been pursued. Unfortunately, many of the molecules identified to date have failed in patient trials due to toxicity, presumably due to consequences of chaperone inhibition in healthy cells (1).

The lack of clinical success of chaperone inhibitors has led researchers to begin to focus on novel ways to modulate chaperone function rather than completely abolish it. One direction has been to explore manipulation of the chaperone code, the posttranslational modifications that regulate chaperone function (2). Another approach has been to explore the role of helper “co-chaperones” and the impact of inhibiting specific cochaperone interactions (Fig. 1). For example, Hsp70 requires a large suite of cochaperones for function. These cochaperones bind unfolded clients and direct them to Hsp70 for folding (1, 3). Additionally, cochaperones such as DNAJA1 are able to activate Hsp70 activity directly by binding Hsp70 and stimulating the Hsp70 ATPase cycle required for chaperone function.

**Figure 1:**
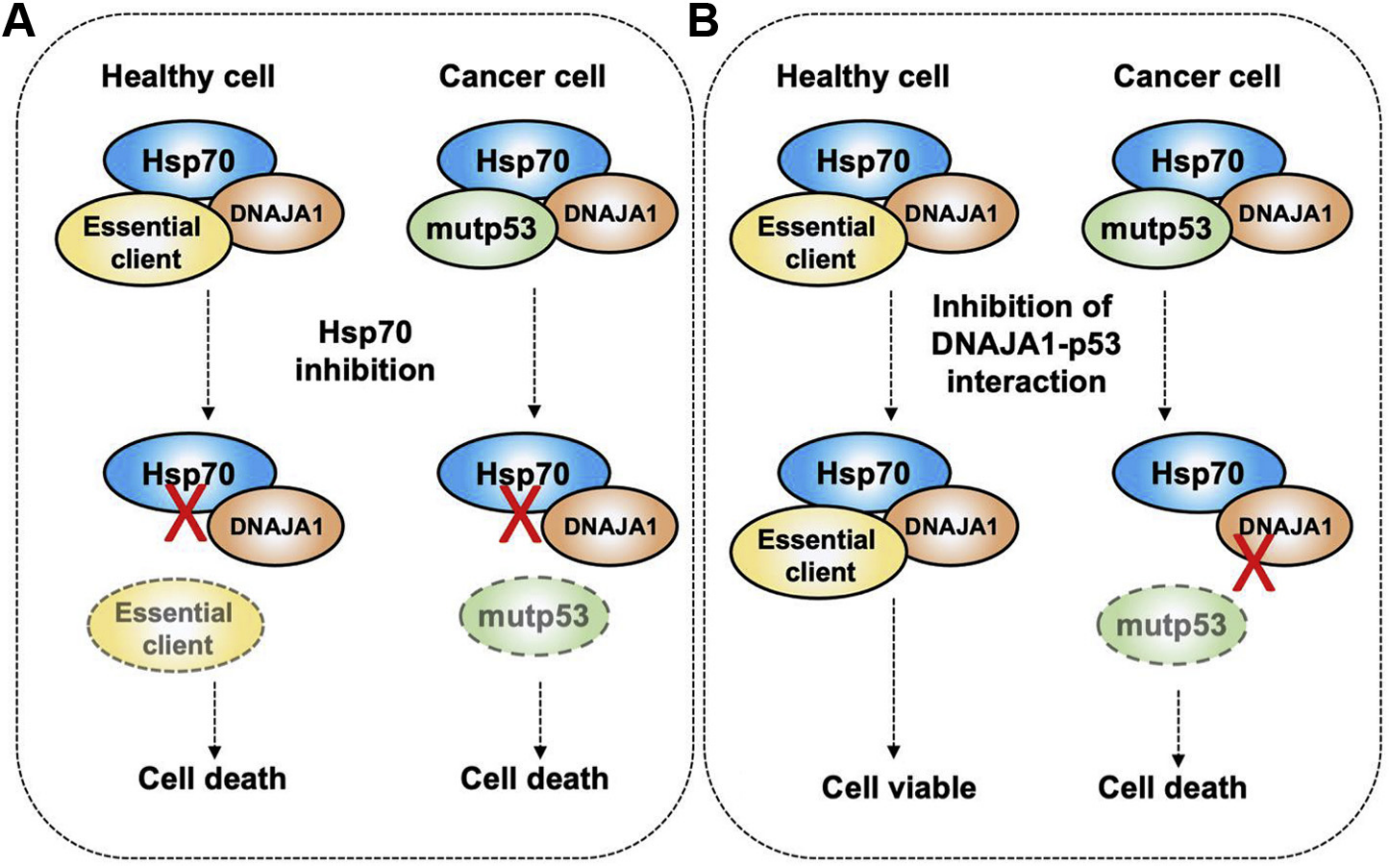
**Inhibition of specific DNAJA1 interactions as an anticancer therapy.***A*, inhibiting Hsp70 function results in global loss of client activity resulting in cell death in both healthy and cancer cells. *B*, disrupting the interaction between DNAJA1 and mutant p53 only impacts cancer cells.

An early indication that Hsp70 cochaperones may play a role in cancer came in 2016 when Parrales and colleagues uncovered a connection between the mevalonate metabolic pathway and the activity and stability of mutant p53, a well-established tumor suppressor (4). Through a series of elegant studies, the team determined that p53 activity was mediated through two cochaperones of Hsp70, DNAJA1 and CHIP (carboxy-terminus of Hsp70 interacting protein). Parralles and coworkers determined mutant p53 is a client of DNAJA1, and their interaction is directly controlled through mevalonate pathway activity, possibly through posttranslational modification of DNAJA1 (4). This finding was confirmed and extended 3 years later, when farnesylation of DNAJA1 at C394 was demonstrated to be critical for DNAJA1–p53 interaction (5). Mutation of DNAJA1 C394 to C394S (a farnesylation-deficient mutant) or treatment of cells with atorvastatin (an inhibitor of farneslyation) promoted p53 degradation (5).

These results raised the question of whether it is possible to directly disrupt the DNAJA1–p53 complex. Tong *et al.* (6) now advance insights into this issue, combining computational modeling, mutation-based *in vitro* work, and mouse models to search for and assess inhibitors of the DNAJA1–p53 interaction. The authors first modeled the interaction between mutant p53 (mutp53^R175H^) and DNAJA1 (6). Despite the numerous structural studies on DNAJA1, no full-length structures have been experimentally determined. In an effort to bypass this issue, the authors utilized multitemplate homology modeling to generate a high-quality DNAJA1 structure. After generating 2000 docking poses of DNAJA1 bound to mutp53^R175H^, 250 low-energy poses were obtained, 10 of which had similar binding modes (6). Using these structures, the authors next looked for druggable interaction pockets at the DNAJA1–p53 interface, identifying four in the glycine/phenylalanine-rich (G/F) region of DNAJA1. Mutation of key residues in the G/F region of DNAJA1 (P84S and K125Q) resulted in destabilization of mutant p53, validating this region of DNAJA1 as an area for targeting as an anticancer therapeutic. The authors then computationally screened a proprietary library of one million molecules. Functional characterization of the top 27 hits identified a small molecular inhibitor, GY1-22, that was able to bind at the mutp53^R175H^-DNAJA1 interface, disrupting the interaction and leading to p53 degradation. This novel small-molecule inhibitor performed well in *in vitro* and *in vivo* mouse studies to inhibit pancreatic cancer cell growth (6).

The findings of Tong *et al*. (6) reinforce the growing body of evidence that cochaperone proteins such as DNAJA1 have excellent potential as anticancer targets. Recent studies have shown DNAJA1 to be important in the activity of other important oncoproteins. For example, DNAJA1 and its yeast counterpart Ydj1 bind, stabilize, and regulate the activity of the ribonucleotide reductase (RNR) complex in both yeast and mammalian cells (7). Inhibition of DNAJA1 function through either knockout *via* CRISPR-CAS9 or *via* the novel small molecule 116-9e developed by the Gestwicki group (8) caused a marked increase in sensitivity to well-characterized RNR inhibitors such as hydroxyurea, triapine, and gemcitabine (7). DNAJA1 also promotes androgen receptor (AR) activity; disruption of the Hsp70–DNAJA1 interaction in castration-resistant prostate cancer cells (CRPC) *via* the small-molecule inhibitor C86 promoted rapid AR degradation (9). Consistent with this observation, C86 significantly decreased average CRPC-derived tumor volume in mouse *in vitro* studies (9).

Of further interest, DNAJA1 may also a play a role in many other signal transduction pathways. Although the complete DNAJA1 interactome has yet to be determined, a recent chemogenomic study revealed that over 30% of a panel of NCI-approved oncology drugs tested showed substantially increased potency in cells lacking DNAJA1 (10). These drugs acted on a variety of pathways including cell cycle progression, DNA damage response and repair, and the cytoskeleton, suggesting a wide range of novel DNAJA1 functions Additionally, these data suggest an untapped space for drug synergy utilizing combinations of DNAJA1 inhibitors with existing approved small-molecule therapeutics. It should be noted, however, that a small but significant number of drugs utilized in the screen were rendered less potent by loss of DNAJA1, possibly explained by DNAJA1 stabilizing proteins whose roles act to inhibit rather than activate specific signal transduction pathways (10). Taken together, these results suggest a rationale for inhibiting specific DNAJA1–client interactions as in the work from Tong and colleagues (6) rather than inhibiting DNAJA1 completely. Future directions may include understanding the range of DNAJA1–client interactions impacted by GY1-22, particularly if several clients share the same DNAJA1 binding site. Additionally, it may be worthwhile understanding if GY1-22 can bind any of the large number of highly similar J-proteins present in cells. Whatever the answer, going forward it may be possible to apply the same strategies by Tong and team to develop many more potent anticancer therapeutics based on destabilizing other selected J-protein–client interactions.

## Conflict of interest

The author declares that he has no conflicts of interest with the contents of this article.
